# Outcomes 10 Years After Implementing an Emergency Department Opt-out Bloodborne Virus Screening Program

**DOI:** 10.1093/ofid/ofaf547

**Published:** 2025-09-11

**Authors:** Liam Townsend, Fiona Herraghty, Seán Brennan, Conor Grant, Wenzhou Wang, Anne Moriarty, Yvonne Lynagh, Lorraine Clancy, Antoinette Power, Brendan Crowley, Suzanne Norris, Darragh Shields, Colm Bergin

**Affiliations:** Department of Infectious Diseases, St James's Hospital, Dublin, Ireland; Department of Clinical Medicine, Trinity College Dublin, Dublin, Ireland; Department of Infectious Diseases, St James's Hospital, Dublin, Ireland; Department of Infectious Diseases, St James's Hospital, Dublin, Ireland; Department of Infectious Diseases, St James's Hospital, Dublin, Ireland; School of Medicine, Royal College of Surgeons Ireland, Dublin, Ireland; Department of Infectious Diseases, St James's Hospital, Dublin, Ireland; Department of Microbiology, St James's Hospital, Dublin, Ireland; Department of Microbiology, St James's Hospital, Dublin, Ireland; Department of Microbiology, St James's Hospital, Dublin, Ireland; Department of Clinical Medicine, Trinity College Dublin, Dublin, Ireland; Department of Microbiology, St James's Hospital, Dublin, Ireland; Department of Clinical Medicine, Trinity College Dublin, Dublin, Ireland; Department of Hepatology, St James's Hospital, Dublin, Ireland; Department of Clinical Medicine, Trinity College Dublin, Dublin, Ireland; Department of Emergency Medicine, St James's Hospital, Dublin, Ireland; Department of Infectious Diseases, St James's Hospital, Dublin, Ireland; Department of Clinical Medicine, Trinity College Dublin, Dublin, Ireland

**Keywords:** HBV, HCV, HIV, screening

## Abstract

**Background:**

Bloodborne viruses (BBV) such as hepatitis B (HBV), hepatitis C (HCV), and HIV pose significant personal and public health risks. Screening and linkage to treating services are important tools in treatment and preventing onward transmission. This study reports outcomes of 10 years of an opt-out BBV screening program in a large urban emergency department (ED).

**Methods:**

Starting in July 2015, ED patients undergoing phlebotomy were offered routine BBV screening. We examine acceptance of screening, characteristics of new diagnoses, and onward linkage to care 10 years after program implementation. We also investigate factors associated with new viremic HCV diagnoses within this cohort to inform future service development.

**Results:**

Over the 10-year period, acceptance of BBV screening among phlebotomized patients was high (81%). There was no significant change in rates of new diagnoses of HIV, HBV, or HCV, but there was a significant reduction in polymerase chain reaction–positive HCV diagnoses. Linkage to care was high (96% HIV, 89% HBV, 95% HCV). Polymerase chain reaction–positive HCV was associated with people who inject drugs and being discharged directly from the ED.

**Conclusions:**

BBV screening in the ED demonstrates sustained acceptability, with a steady rate of new diagnoses detected. It provides high levels of linkage to care. It also identifies active HCV within a population of people who inject drugs attending the ED who are discharged directly without needing admission.

## Teaser

Bloodborne virus screening allows for early diagnosis, linkage to care, and prevention of transmission. A decade after its introduction in our emergency department, we show high levels of acceptability and care linkage and identify populations in need of outreach screening interventions.

## Graphical Abstract

**Graphical Abstract:**
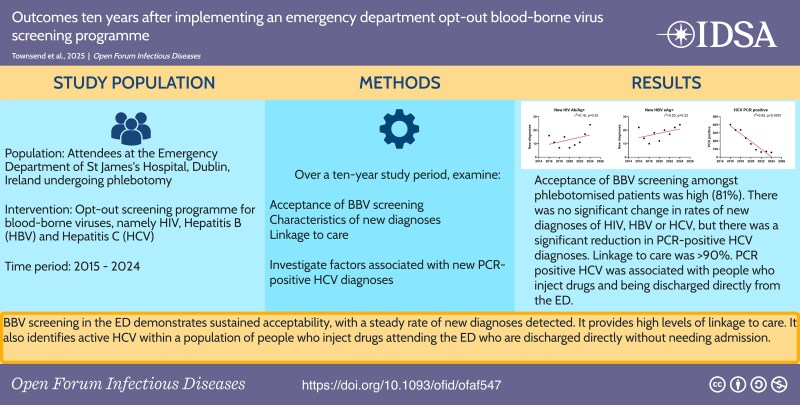
This graphical abstract is also available at Tidbit: https://tidbitapp.io/tidbits/outcomes-ten-years-after-implementing-an-emergency-department-opt-out-blood-borne-virus-screening-programme?utm_campaign=tidbitlinkshare&amp;utm_source=ITP

Bloodborne viruses (BBV), in particular HIV, hepatitis B virus (HBV), and hepatitis C virus (HCV), cause significant morbidity and mortality. Early detection and prompt treatment reduce this burden, as well as preventing onward transmission [1, 2]. A significant proportion of infected individuals are thought to be unaware of their diagnosis [3–5]. Increasing access to testing, including opportunistic testing of individuals at point of access to healthcare services, will increase diagnostic yield and linkage to care [6].

Lack of awareness by both healthcare professionals and patients, as well as stigma, have been associated with delays in BBV diagnosis [7]. Screening patients undergoing phlebotomy in emergency departments (ED) using an opt-out system provides an opportunity to test large numbers of patients, including those who do not routinely access healthcare through scheduled care. This allows for detection of new diagnoses as well as relinkage to care for patients who may have been lost to follow up, and reduces barriers to marginalized populations [8, 9].

Opt-out testing remains far from universal across healthcare systems. Many countries, including the United Kingdom, have only commenced national screening in recent years [10]. This is in contrast to our center, where a pilot opt-out screening system for BBVs was initiated in the ED in 2014 [11]. This demonstrated feasibility and high levels of acceptability amongst patients [12]. The Emergency Department Viral Screening (EDVS) Program at St James's Hospital was subsequently launched in the summer of 2015. Here, we report on the first 10 years of this program, describing acceptability, the changes in the patient cohort over time, linkage to care, and the impact of COVID-19 restrictions on care linkage. We investigate predictors associated with new diagnoses of polymerase chain reaction (PCR)-positive HCV and outline how the testing model needs to adapt to continue to diagnose these patients.

## METHODS

### Clinical Setting and Protocol

The EDVS program offers screening to all adult patients with intact decision-making capacity who undergo phlebotomy as part of routine care in the ED of St James's Hospital, Dublin, Ireland. This began as a pilot study in 2014. At that point, patient information leaflets were provided and verbal consent was obtained from participants. Following the success of the pilot, the EDVS program has been incorporated into standard of care in the ED since July 2015 and is delivered as part of routine care by ED staff. An interim review of the program had been planned after 5 years. Because of the intercurrent COVID-19 pandemic and the associated changes in workload priorities, this review was deferred to the 10-year timepoint, and this study represents the first evaluation and potential redesign of the EDVS program. An additional serum tube for screening is taken unless patients explicitly opt out. An EDVS order option has been added to the electronic health record and is included in routine care bundles for all ED attendees (eg, basic medical and surgical laboratory panels). The EDVS order option prints a label for testing of combined HIV antibody/antigen, HCV antibody, and HBV surface antigen on a single serum sample bottle. Under the current EDVS model, patients are informed that BBV testing is part of the standard of care. Patients must explicitly decline should they prefer screening not to be performed. In the event that patients declines, their choice is not documented but is inferred from the absence of an EDVS order. Patients do not incur any costs associated with these tests. St James's Hospital is a large tertiary referral center serving a predominantly socially deprived urban catchment area. EDVS screening was not offered to patients who were not undergoing phlebotomy as part of routine ED care. Because of the large number and high turnover of staff performing phlebotomy in the ED, including doctors, nurses, and phlebotomists, there was no requirement to check when the most recent EDVS screen was performed before a screen being repeated to ensure total coverage and uptake remained high. Additionally, the hospital serves a catchment area with high rates of behaviors associated with BBV acquisition, providing a rationale for repeat screening [13].

Screening tests were performed on the Abbott Alinity *I*, with the Alinity HIV Ag/Ab combination reagent used for HIV screening, Alinity HBsAg Qual II for hepatitis B surface antigen (HBsAg) screening, and Alinity anti-HCV and Innotest HCV Ab IV for hepatitis C antibody screening and confirmation (all Abbott Laboratories, Illinois, USA). Biomerieux Vidas HIV5 (Biomerieux, Marcy-L’Étoile, France) and Bio-Rad Geenius HIV 1/2 (Bio-Rad Laboratories, California, USA) confirmatory assay were used for confirmation and typing of HIV, with Alinity anti-HepBc for HBV core antibody testing. PCR for HCV RNA was performed on all samples with positive HCV antibody, unless the patient had undergone EDVS screening within the preceding 3 months, as identified by laboratory scientists. The testing cascade is shown in Supplementary Figure 1.

The funding for the EDVS program includes a clinical nurse specialist in an EDVS liaison role. Clinical leads within the infectious diseases, emergency medicine, and virology departments formed a steering committee, which meets every 6 months. All positive results are reported to the EDVS liaison nurse. For patients with positive results who were admitted via the ED, the EDVS nurse would make contact with them during their inpatient stay, perform confirmatory tests, perform a FibroScan for patients with HBV or HCV, and refer them to subsequent outpatient care. For patients with positive result who were discharged either directly from ED or before initial screening result becoming available, the EDVS liaison nurse would contact them via telephone and text message. In the case where no response was obtained, a letter is sent to their address, inviting them to attend the infectious diseases day ward for assessment. Day ward assessment is identical to inpatient assessment, with screening, staging, and onward referral performed.

### Data Collection

Data were extracted from the ED interactive whiteboard system and the hospital electronic patient record. Data collection was from inception of the EDVS program in July 2015 until December 2024. Total ED attendances, number of patients undergoing phlebotomy, and number of EDVS screens carried out during this period were recorded. Positive EDVS results over the study period were recorded in an electronic database, maintained by a dedicated EDVS liaison nurse. Results were recorded as total positive tests, unique patient encounters, and new diagnoses. Additional data collected included demographics (sex, age, country of origin), PCR results (where relevant), and linkage to care. These were obtained through a combination of data from the electronic health record and the first visit with the EDVS liaison nurse. Likely mode of acquisition, such as person who injects drugs, gay, bisexual, and men who have sex with men, heterosexual, or country of high prevalence were obtained in a similar manner. Housing status (either housed or homeless), whether the patient had a primary care physician, and onward disposition from ED, which was classified as either referred for admission or discharged directly by ED, was recorded. Attending ED as a primary consequence of substance misuse was also recorded. This incorporated accidental overdoses, intoxication, and any transient behavioral disturbance as a result of substance misuse. These data were derived from the presenting complaint and patient history on the electronic patient record. Patients with medical issues as a complication of substance misuse, beyond the expected spectrum of substance misuse, were not included in this category (eg, patients with thrombosis secondary to person who injects drugs or psychosis in the setting of drug misuse were not categorized as directly due to substance misuse). Linkage to care was defined as attending a follow-up appointment with the EDVS liaison nurse, and if confirmatory test demonstrated an active infection, attendance at a relevant clinic within 3 months of positive result.

### Statistical Analysis

Descriptive statistics are reported as means with standard deviations (SD) and medians with interquartile ranges (IQR), as appropriate. For data analysis purposes, each complete calendar year was included as a period time. The initial 6 months from July 2015 to December 2015 are omitted from data visualization. Changes in absolute number of diagnoses per year and changes in linkage to care were assessed using the Cochran-Armitage test with Montecarlo correction, whereas changes in proportions over years were calculated using linear regression. Given that some patients presented to ED multiple times over the 10 years of the program and underwent multiple screening tests, testing results as a function of total positive tests, unique patient encounters, and new diagnoses were assessed. Univariate analysis was used to assess differences in patients with positive HBV, HCV, and HIV tests in the first full calendar year of the program (2016) and the most recent full calendar year (2024), as well as differences in PCR-positive HCV infection. Multivariable logistic regression was used to assess factors associated with PCR-positive HCV infection, with all significant variables on univariable analysis included in the model. Models were examined for multicollinearity by computing variance inflation factors. All analysis was performed using Stata version 18.0 (Stata Statistical Software).

## RESULTS

### Screening Uptake and Positive Results

There was an average of 49 000 ED attendances per year over the 10-year study period from 2015 to 2024. Phlebotomy was performed in approximately 62% of attendees. Of those undergoing phlebotomy, acceptance of viral screening was 81%, with more than 200 000 screens performed over the study period. Viral screening was more likely to be accepted by younger patients (*z* = 10.2, *P* < .0001), whereas there were no sex differences in uptake.

**Figure 1.**
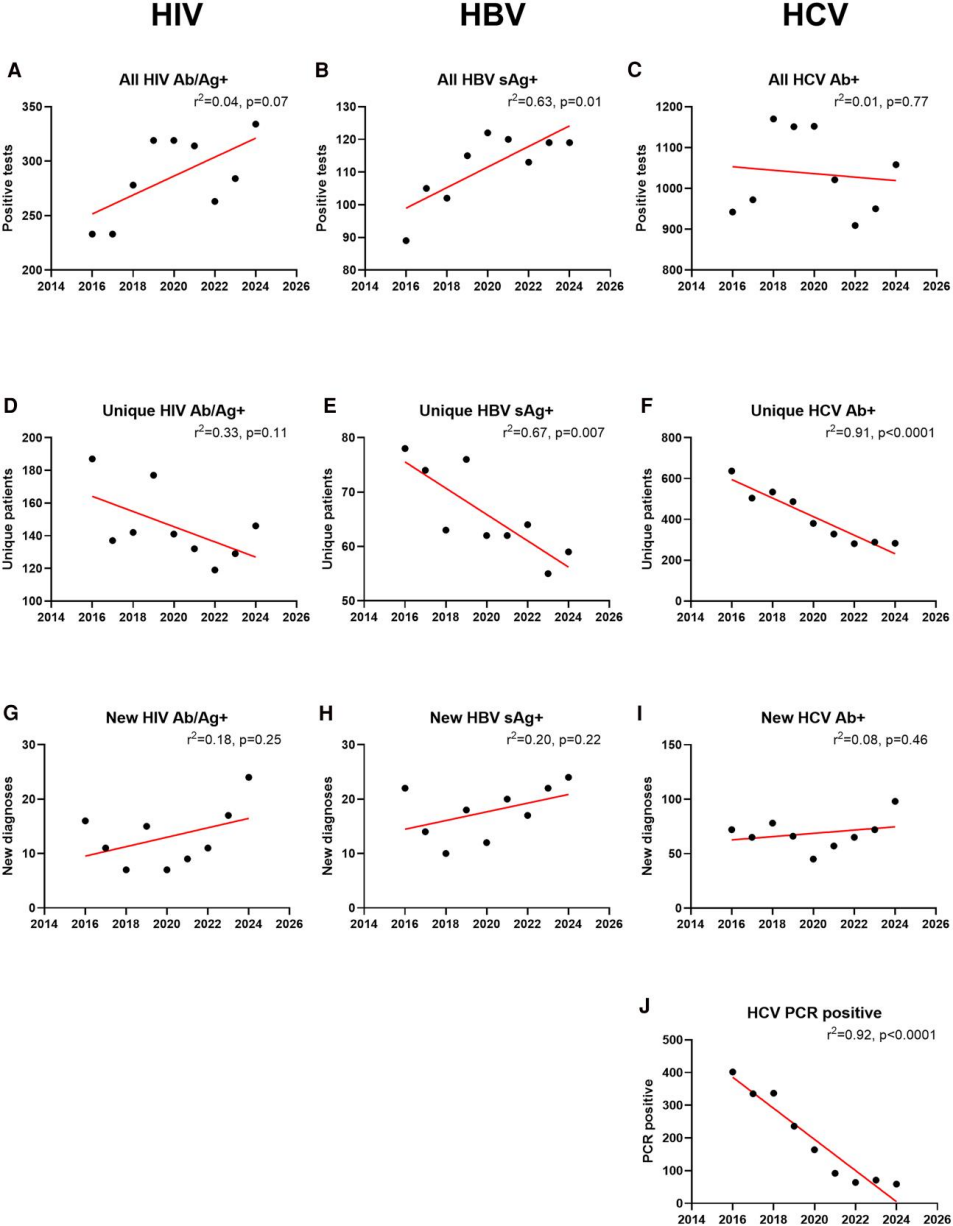
Changes in results over 10 years. Rates of positive HIV Ab/Ag, HbsAg, and HCV Ab (*A–C*), rates of unique patients with positive tests (*D–F*), rates of new diagnoses (*G–I*) and rates of HCV PCR positivity (*J*) from 2016 to 2025.

There were 2663 positive HIV tests in 1384 individuals, with 114 being new diagnoses. There were 1035 positive HBsAg results in 624 individuals, accounting for 156 new diagnoses, whereas positive HCV antibody tests were recorded 9764 times in 4105 individuals. A total of 565 patients with positive HCV antibody had no prior knowledge of HCV infection at any time in the past, nor had they undergone prior treatment. HCV PCR was positive in 1357 (14%) instances of positive tests. The rates of positive results over the course of then 10 years was highly variable (Figure 1*A*—*C*). However, following exclusion of repeat visits by individuals, there was a steady decrease in unique patients identified via viral screening over the years, with significant declines in the rate of new HBV (Figure 1*E*) and HCV (Figure 1*F*) attendees. Rates of new diagnoses of HIV, Hepatitis B sAg + and positive HCV antibody in patients who were unaware of their status remained steady (Figure 1*G*—*I*). However, new diagnoses of active HCV infection reduced over time, with a significant decline in HCV PCR-positive attendees (Figure 1*J*).

### Characteristics of Attendees With Positive HIV and HBV Results Over Time

To identify changes in the patterns of attendance and diagnostics over time, the characteristics of unique patients testing positive for HIV, HBV, and HCV in the first full calendar year of the screening program (2016) were compared with those from the most recent full calendar year (2024). The majority of individuals with positive viral screens were monoinfected. There was a large drop in HIV-positive participants who also were PCR-positive HCV over the years, from 40% in 2016 to 14% in 2024 (Supplementary Figure 2).

There was a significant reduction in patients born in Ireland testing positive for HIV and HBV over time compared to those born elsewhere. Patients with positive HBsAg in 2024 were significantly older than those testing positive in 2016. Complete details of the changes over time are shown in Table 1.

**Table 1.** Characteristics of Patients With Positive HIV and HBV in 2016 and 2024

	HIV			HBV		
	2016 (n = 187)	2024 (n = 146)	Statistic	2016 (n = 78)	2024 (n = 59)	Statistic
Age, years; median (IQR)	40 (14)	42.5 (16.5)	*z* = −1.26, *P* = .21	40 (20)	46 (16)	*z* = −1.97, *P* = .04
Sex, female; n (%)	60 (32)	48 (33)	Χ^2^ = 0.02, *P* = .88	28 (36)	17 (29)	Χ^2^ = 0.76, *P* = .38
Region of origin; n (%)						
Ireland	128 (68)	69 (47)	*r* ^2^ = 0.08, *P* < .0001	22 (28)	5 (8)	*r* ^2^ = 0.12, *P* < .0001
South America	18 (10)	22 (15)	…	9 (12)	1 (2)	…
Sub-Saharan Africa	23 (12)	18 (12)	…	14 (18)	7 (12)	…
Eastern Europe	9 (5)	4 (3)	…	3 (4)	4 (7)	…
Western Europe	3 (2)	2 (1)	…	16 (21)	19 (32)	…
Unknown	3 (2)	19 (13)	…	10 (13)	13 (22)	…
Other	3 (2)	12 (8)	…	4 (5)	10 (17)	…
Risk						
PWID	78 (42)	43 (29)	*r* ^2^ = 0.001, *P* = .50	8 (10)	4 (7)	*r* ^2^ = 0.01, *P* = .21
Heterosexual	13 (7)	33 (23)	…	4 (5)	2 (3)	…
gbMSM	64 (34)	50 (34)	…	35 (45)	26 (44)	…
COHP	15 (8)	0 (0)	…	16 (21)	9 (15)	…
Unknown	12 (6)	20 (14)	…	15 (19)	18 (31)	…
Vertical	5 (3)	0 (0)	…	…	…	…
New diagnosis; n (%)	16 (9)	19 (13)	Χ^2^ = 1.93, *P* = .17	22 (28)	21 (36)	Χ^2^ = 2.67, *P* = .10
Linked to care, yes; n (%)	181 (97%)	140 (96%)	Χ^2^ = 0.19, *P* = .66	67 (85)	43 (79)	Χ^2^ = 3.16, *P* = .08

All unique patients with positive antigen included. Subsequent visits by the same patient were not included in analysis. Groups compared with Wilcoxon rank-sum, chi-squared, or analysis of variance, as appropriate.

Abbreviations: COHP, country of high prevalence; gbMSM, gay, bisexual and men who have sex with men; PWID, person who injects drugs.

### Linkage to Care

**Figure 2.**
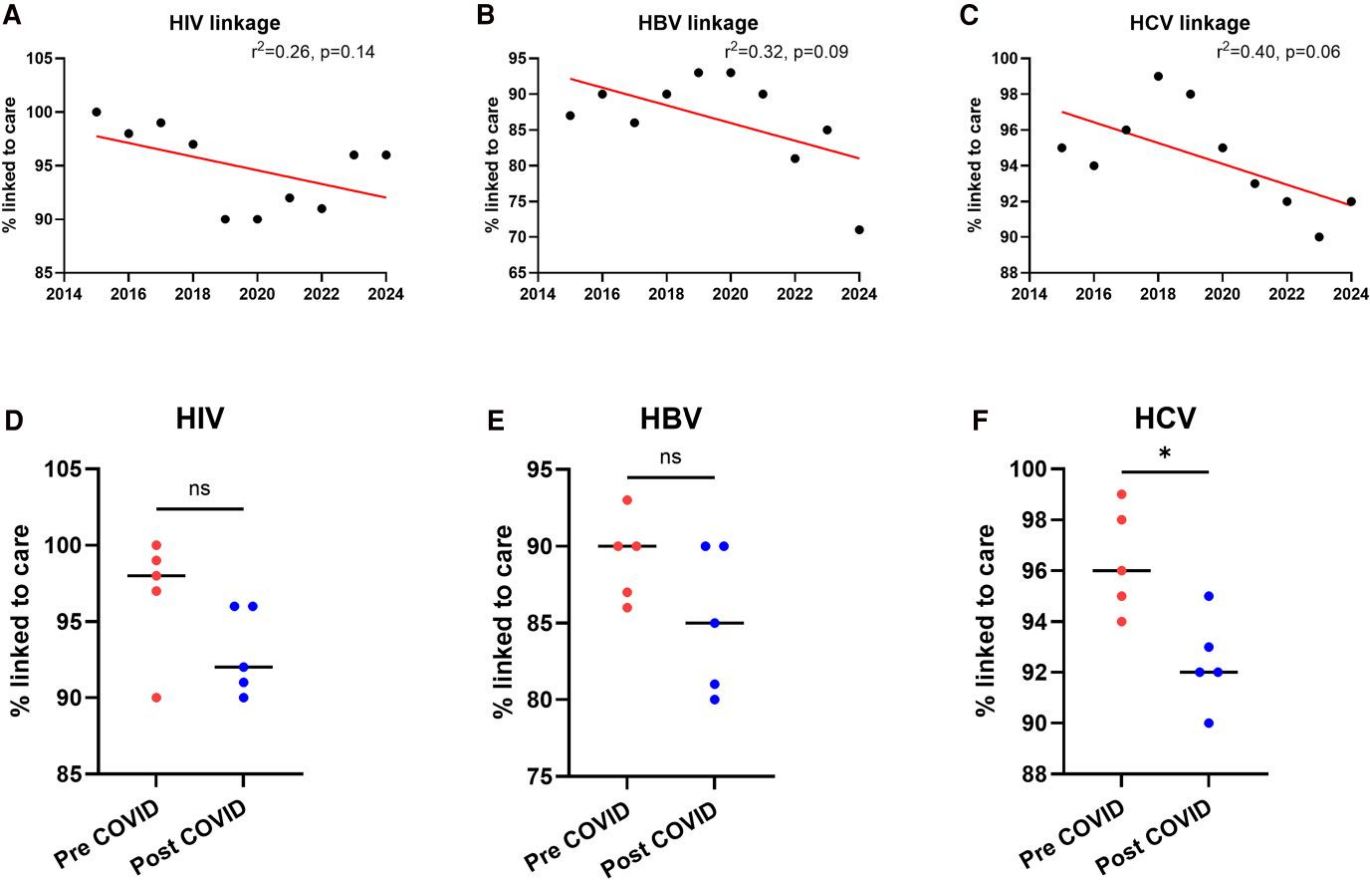
Linkage to care over time. Linkage to care over the 10 years of EDVS for **(***A***)** HIV, **(***B***)** HBsAg+, and **(***C***)** hepatitis C PCR-positive patients. Linkage with care during pre-COVID-19 and during/post-COVID-19 restrictions shown in *D—F*. Cochrane-Armitage test with Montecarlo correction and Wilcoxon rank sum used to assess significance.

Linking individuals with a positive screening test to care is a key metric for any screening program. Each positive test in a person not linked to care was considered an opportunity for linkage. Patients with more than 1 positive screening event over time were considered as separate data points for linkage to care. There were no significant changes in linkage to care across the 10 years for any of the BBVs tested, with median 96% linkage for HIV, 89% for HBV, and 95% for HCV (Figure 2*A*, 2*B*, 2*C*). However, there was an overall trend in reduced linkage to care over the course of the EDVS program. A secondary analysis of linkage to care before (2015–2019) and after (2020–2024) COVID-19 restrictions was performed. This demonstrated a significant drop in linkage to care for HCV (Figure 2*F*), with no change in HIV or HBV linkage (Figure 2*D*, *E*). Patients with new diagnoses picked up via EDVS were more likely to be successfully linked to care then those with known diagnoses who were not already in routine follow up. All patients with HCV and HBV underwent a FibroScan to evaluate degree of liver fibrosis. The median FibroScan score in the new PCR-positive HCV patients was 6.3 kPa (IQR 5–8.4), with 68 patients having a score ≥8 kPa. The median FibroScan score in new HbsAg+ patients was 5.4 kPa (IQR 4.6–6.4), with 16 patients having a score ≥8 kPa.

### Characteristics of New HCV Diagnoses

While new diagnoses of antibody-positive HCV are relevant in evaluating the epidemiology and patterns of infection, individuals with newly diagnosed PCR-positive HCV infection represent an important cohort, as they can avail of curative treatment and reduce the risk of onward transmission. The significant drop in linkage to care for PCR-positive HCV patients was investigated further. The characteristics of patients with a new diagnosis of antibody-positive HCV infection during 2016 and 2024 and subsequent PCR positivity were evaluated to assess changes in disease epidemiology. There were n = 71 new HCV antibody-positive diagnoses in 2016, of whom n = 38 (54%) were PCR positive, compared to n = 48 in 2024, of whom n = 38 (79%) were PCR positive. Within the new diagnoses of HCV across both 2016 and 2024, PCR-positive disease was associated with younger age, male sex, active intravenous drug use, having no fixed abode, and not requiring onward referral for admission from the ED, as well as having had a prior EDVS screen (Supplementary Table 1). Predictors of new PCR-positive HCV infection were further investigated using a logistic regression model, including all significant variables on univariate analysis. Active intravenous drug use remained a significant predictor, while not requiring onward referral for admission and being discharged directly from the ED was also significantly associated with having a positive HCV PCR (Table 2).

**Table 2.** Factors Associated With a New Diagnosis of PCR-positive HCV

	Odds Ratio (95% CI)	*P* Value
Age	0.98 (.94–1.01)	.19
Sex, female	0.46 (.17–1.22)	.12
PWID	3.01 (1.15–7.94)	.03
Homeless	2.29 (.84–6.22)	.10
Referred for admission	0.35 (.14–.89)	.03
First EDVS test	0.46 (.19–1.14)	.09

Multivariable logistic regression for n = 76 patients with a new diagnosis of PCR-positive HCV, with all variables included in the model shown.

Abbreviations: CI, confidence interval; EDVS, emergency department viral screen; HCV, hepatitis C virus; PCR, polymerase chain reaction; PWID, person who injects drugs.

## DISCUSSION

This study demonstrates the feasibility and sustained success of an ED BBV screening program over a 10-year period. We show consistently high uptake of screening by patients and high rates of linkage to care. We describe the changing epidemiology of HIV, HBV, and HCV infection and, importantly, we identify patient groups who would benefit from additional screening interventions to capture active infection, in particular individuals whose only interaction with healthcare systems may be the ED.

More than 4 of 5 patients undergoing phlebotomy in the ED accepted BBV screening, with more than 200 000 screens performed over the 10-year period. The number of unique patients receiving positive diagnoses reduced over the past decade. This is unsurprising, given that the initial years represent lead-in time for the program and likely capture a large proportion of individuals who were frequently attending the ED prior to screening introduction. Despite this reduction in unique attendees, there was no significant change in new diagnoses of HIV, HBV or HCV over the years. This emphasizes the importance of screening programs, as capturing new diagnoses and linking them to care will help achieve eradication targets for BBV [14, 15]. The significant reduction in PCR-positive disease seen over the course of the EDVS program may reflect the influence of direct-acting antiviral therapy [16, 17]. However, this was not directly assessed so causation cannot be inferred. The large number of repeat screens in a population who already have a known diagnosis identifies an opportunity for diagnostic stewardship. Increased education within the ED in addition to prompts within the electronic health record may reduce unnecessary repeated testing.

The demographics and epidemiology of patients with positive EDVS screening results has evolved over the 10-year period, reflecting wider changes in Irish society. Although gay, bisexual, and men who have sex with men remains the largest risk factor for HIV acquisition, there has been a reduction in people who inject drugs and increase in heterosexual acquisition risk. The effect of migration is also apparent, with a significant reduction in Irish patients testing positive for HIV. While this is similar to demographic changes seen in the overall HIV population in Ireland, the increase in patients with unknown region of origin limits interpretation of these results [18]. In contrast, the ethnic and risk background for new positive HCV antibody results has remained static, with an increase in age at time of diagnosis. Although absolute number of new positive HCV antibody has reduced, there was a significant increase in the proportion of patients for whom this was a new diagnosis.

Linkage to care has remained high across all 3 infections over the years, and was higher than those reported elsewhere [19, 20]. Despite concerns that COVID-19 restrictions and changes in practice, with a reduction in in-person reviews, would lead to a drop in linkage to care, there were no significant changes in HIV and HBV linkage [21–23]. The significant decrease in HCV linkage therefore may not be a direct effect of COVID-19 restrictions. Examining the HCV PCR-positive cohort in more details demonstrated a group of patients with active intravenous drug use, who were significantly less likely to require hospital admission than those with PCR-negative disease. There was also an increased proportion of PCR-positive patients who had previous negative EDVS tests. The ED is well recognized as being the primary site of healthcare for marginalized groups [24, 25].

This suggests that active HCV infection is continuing to propagate amongst injection drug users who use healthcare systems in a different manner to other groups, in particular attending tertiary centers for nonurgent care rather than primary care providers [26, 27]. This highlights the need to have a screening program at first contact with healthcare systems, rather than after admission, where the positive screening rates have previously been shown to be low [28]. The significantly higher proportion on univariate analysis of PCR-positive individuals who had a prior negative EDVS test also emphasizes the need for ongoing rescreening in at-risk groups. This cohort remains challenging to engage in screening and sustained engagement with care, and likely require an alternative to traditional care models [29]. This may involve mobile community treatment units, dedicated keyworkers, addiction support, and test and treat policies. These have been trialed previously in HCV, with variable rates of success [30–32]. However, lessons may be learnt from the success of outreach programs for HIV [33, 34].

There are several limitations to this study. Approximately 20% of patients opted out of BBV screening. Additionally, 40% of ED attendees did not undergo phlebotomy as part of routine care and were therefore not eligible for EDVS screening. This cohort is particularly important, given that PCR-positive HCV patients were more likely to be discharged directly from ED and would likely be well-represented in this nonphlebotomized cohort. While linkage to care is recorded, data on retention in care or adherence to prescribed therapies are not recorded. This will be an area for future study.

Overall, this study demonstrates the sustained success of an opt-out programme for BBV screening in emergency departments, and can strengthen the case to implement such programmes in other jurisdictions. It also provides a template for the establishment of modified screening programmes to test and treat vulnerable and socially-excluded patient populations.

## Supplementary Material

ofaf547_Supplementary_Data

## Supplementary Data

Supplementary materials are available at *Open Forum Infectious Diseases* online. Consisting of data provided by the authors to benefit the reader, the posted materials are not copyedited and are the sole responsibility of the authors, so questions or comments should be addressed to the corresponding author.
