# Supplementary material for: Outcomes 10 Years After Implementing an Emergency Department Opt-out Bloodborne Virus Screening Program
Source: Open Forum Infect Dis. 2025 Sep 11;12(9):ofaf547. doi: 10.1093/ofid/ofaf547 (PMC12456172; doi:10.1093/ofid/ofaf547)
Supplement: ofaf547_Supplementary_Data [file ofaf547_supplementary_data.zip › Supplemental material.docx]

**Supplemental Figure 1: Laboratory testing algorithm**

**
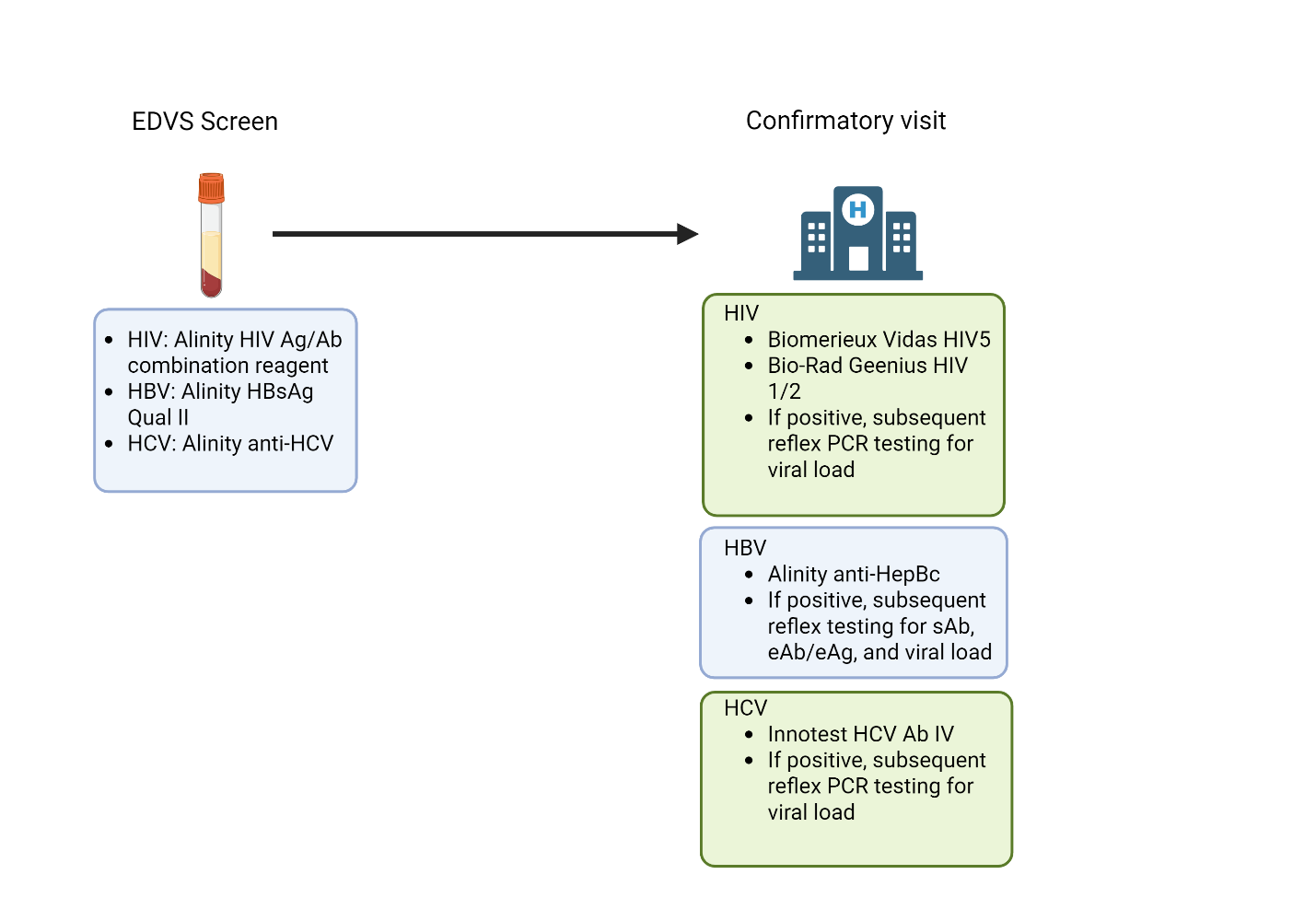
**

Supplemental Figure 1: Initial screening tests as part of EDVS panel, with subsequent testing cascade for positive screens. Viral load/PCR not repeated if previously performed within the preceding three months. sAb = surface antibody, eAb = e antibody, eAg = e antigen

**Supplemental Figure 2: Co-infection status of cohort**


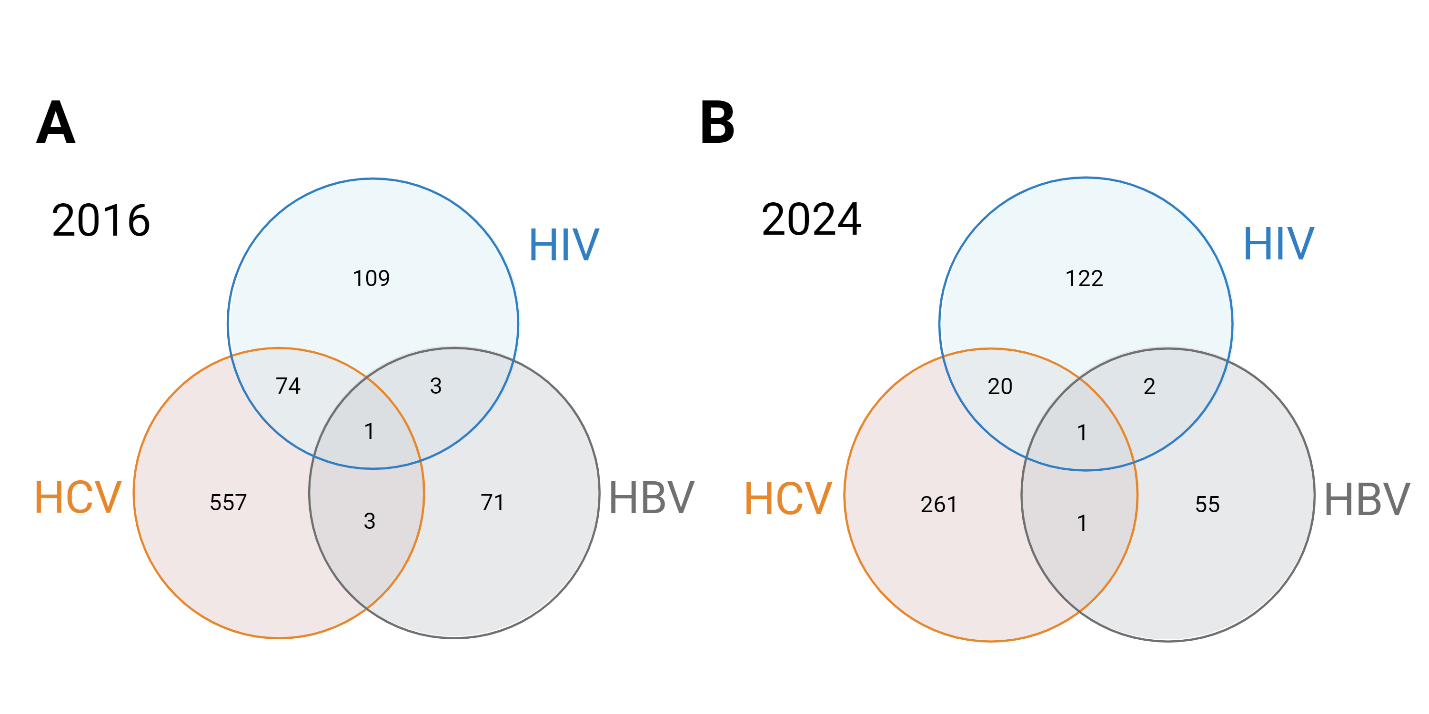


Supplemental Figure 2: Distribution of HIV, PCR-positive HCV and HBV surface antigen-positive infection and co-infection in (**A**) 2016 and (**B**) 2024. Significant reduction in HIV-HCV co-infection from 2016 – 2024 (40% to 14%, Χ^2^=4.39, p=0.04). Remainder non-significant.

**Supplemental Table 1: Characteristics of new diagnoses of PCR-positive HCV infection**

|  | PCR positive (n=76) | PCR negative (n=43) | Statistic |
| --- | --- | --- | --- |
| Age, years; median (IQR) | 38 (15) | 46 (28) | *z*=2.51, p=0.01 |
| Sex, female; n (%) | 17 (22) | 17 (40) | Χ^2^=3.97, p=0.04 |
| Ethnicity, Irish; n (%) | 57 (75) | 33 (77) | Χ^2^=0.01, p=0.93 |
| Risk, PWID; n (%) | 60 (79) | 20 (47) | Χ^2^=13.06, p<0.0001 |
| Homeless; n (%) | 31 (41) | 9 (21) | Χ^2^=4.53, p=0.03 |
| No Primary Care Physician; n (%) | 42 (32) | 13 (30) | Χ^2^=1.43, p=0.23 |
| Referred for Admission; n (%) | 24 (32) | 22 (51) | Χ^2^=4.44, p=0.04 |
| Substance misuse; n (%) | 22 (29) | 7 (16) | Χ^2^=2.39, p=0.12 |
| First EDVS test; n (%) | 38 (54) | 7 (15) | Χ^2^=5.10, p=0.02 |

All unique patients included. Subsequent visits by the same patient were not included in analysis. New diagnoses from 2016 and 2024 included in analysis. PWID=person who injects drugs. EDVS=Emergency department viral screen. Groups compared with Wilcoxon rank sum and Chi-squared, as appropriate
