# Supplementary figures and images for: Outcomes 10 Years After Implementing an Emergency Department Opt-out Bloodborne Virus Screening Program
Source: Open Forum Infect Dis. 2025 Sep 11;12(9):ofaf547. doi: 10.1093/ofid/ofaf547 (PMC12456172; doi:10.1093/ofid/ofaf547)

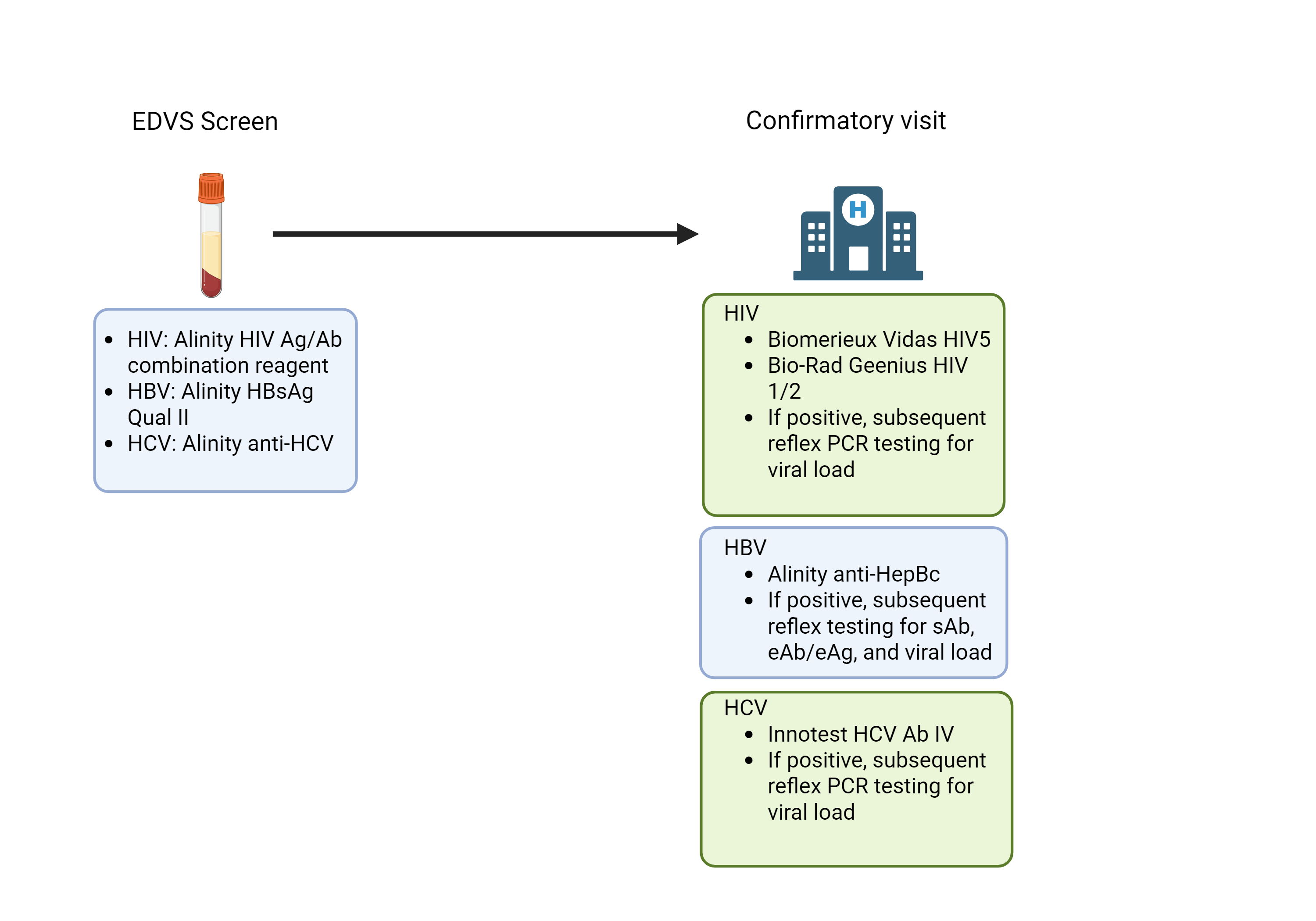

Supplement: ofaf547_Supplementary_Data [file ofaf547_supplementary_data.zip › Supplemental Figure 1.tif]

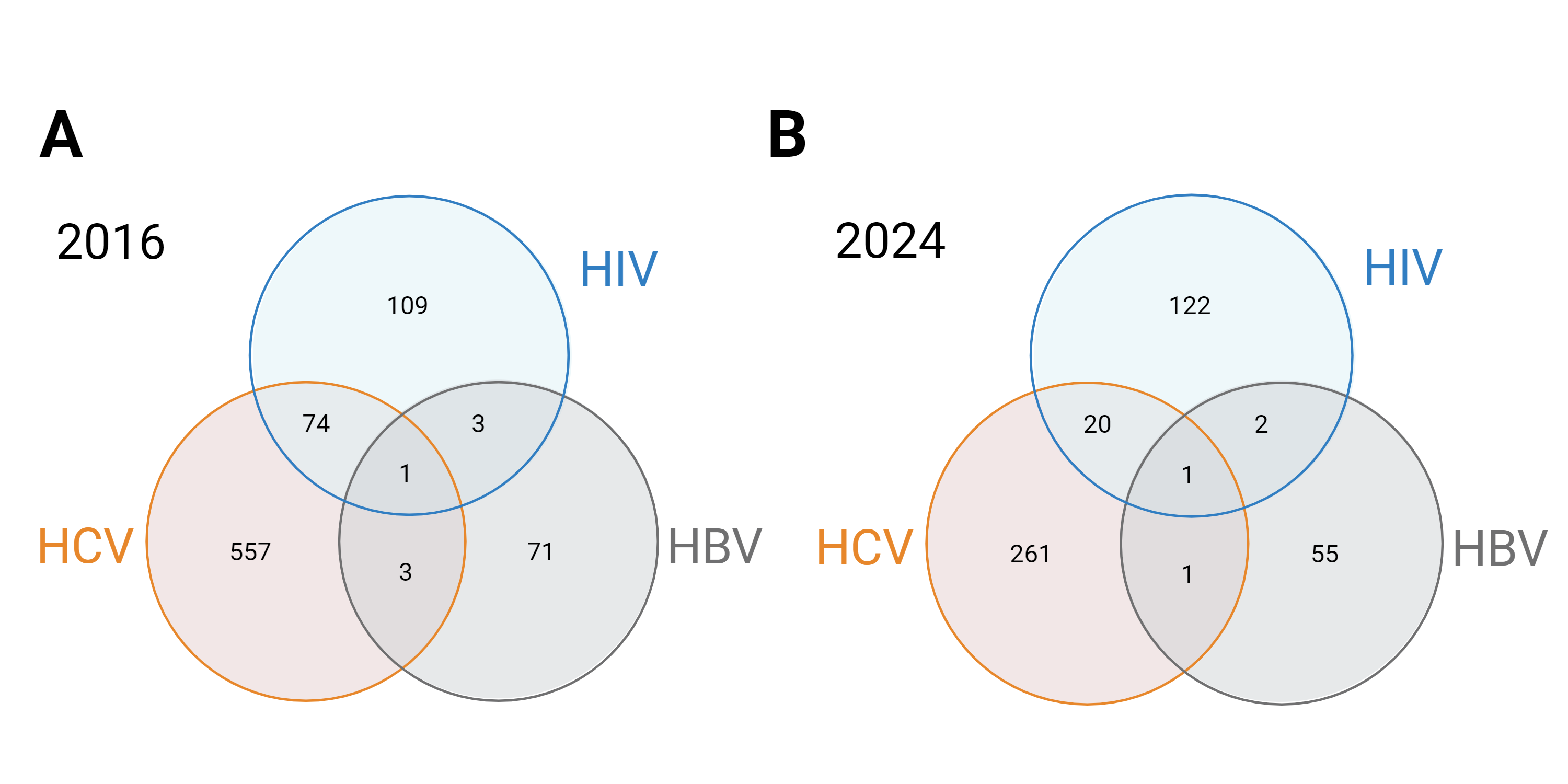

Supplement: ofaf547_Supplementary_Data [file ofaf547_supplementary_data.zip › Supp Figure 2.png]
